# Multistate analysis of prospective Legionnaires’ disease cluster detection using SaTScan, 2011–2015

**DOI:** 10.1371/journal.pone.0217632

**Published:** 2019-05-30

**Authors:** Chris Edens, Nisha B. Alden, Richard N. Danila, Mary-Margaret A. Fill, Paul Gacek, Alison Muse, Erin Parker, Tasha Poissant, Patricia A. Ryan, Chad Smelser, Melissa Tobin-D’Angelo, Stephanie J. Schrag

**Affiliations:** 1 Centers for Disease Control and Prevention, Atlanta, Georgia, United States of America; 2 Colorado Department of Public Health and Environment, Denver, Colorado, United States of America; 3 Minnesota Department of Health, St. Paul, Minnesota, United States of America; 4 Tennessee Department of Health, Nashville, Tennessee, United States of America; 5 Connecticut Department of Public Health, Hartford, Connecticut, United States of America; 6 New York State Department of Health, Albany, New York, United States of America; 7 California Emerging Infections Program, Oakland, California, United States of America; 8 Oregon Health Authority, Portland, Oregon, United States of America; 9 Maryland Department of Health, Baltimore, Maryland, United States of America; 10 New Mexico Department of Health, Santa Fe, New Mexico, United States of America; 11 Georgia Department of Public Health, Atlanta, Georgia, United States of America; Imperial College London, UNITED KINGDOM

## Abstract

Detection of clusters of Legionnaires’ disease, a leading waterborne cause of pneumonia, is challenging. Clusters vary in size and scope, are associated with a diverse range of aerosol-producing devices, including exposures such as whirlpool spas and hotel water systems typically associated with travel, and can occur without an easily identified exposure source. Recently, jurisdictions have begun to use SaTScan spatio-temporal analysis software prospectively as part of routine cluster surveillance. We used data collected by the Active Bacterial Core surveillance platform to assess the ability of SaTScan to detect Legionnaires’ disease clusters. We found that SaTScan analysis using traditional surveillance data and geocoded residential addresses was unable to detect many common Legionnaires’ disease cluster types, such as those associated with travel or a prolonged time between cases. Additionally, signals from an analysis designed to simulate a real-time search for clusters did not align with clusters identified by traditional surveillance methods or a retrospective SaTScan analysis. A geospatial analysis platform better tailored to the unique characteristics of Legionnaires’ disease epidemiology would improve cluster detection and decrease time to public health action.

## Introduction

Legionnaires’ disease, a severe pneumonia typically caused by inhalation of aerosolized water containing *Legionella* bacteria, is responsible for more than 7,400 reported illnesses annually in the United States (U.S.) [1, 2] though this likely underestimates the true burden of disease [3]. Clusters, which more than tripled from 2009–2017 [4], can be large, resulting in significant morbidity and mortality [5–8], or small [9, 10], which makes rapid detection challenging. Additionally, environmental sources implicated in clusters include cooling towers, which can transmit bacteria for miles [11], showers or other potable water sources, whirlpool spas, and decorative water features such as fountains [12]. Identification of an exposure source often requires reviewing potential environmental exposures during the ‘incubation period’ (defined as the 10–14 days before disease onset) and comparing bacterial isolates collected from environmental and clinical samples. Clinical isolates have become increasingly rare since the widespread uptake of urinary antigen testing. A lack of clinical isolates, failure to identify epidemiologic links among cases, and a large number of possible environmental sources can complicate cluster detection. Thus, the true burden of sporadic (cases with no known epidemiologic or molecular links) versus cluster-associated Legionnaires’ disease is not fully understood.

Legionnaires’ disease clusters can have widely varying characteristics. Those associated with travel typically have a readily identifiable common exposure (such as a single hotel), simplifying their detection. Most travel clusters involve two or three cases, which is enough to trigger a public health investigation [13]. In contrast, community clusters often do not begin with a known shared exposure, making them more difficult to detect and investigate. In the absence of factors that could simplify identification such as a sharp spike in cases, unusual geographic clustering, or a very low baseline of disease, these clusters can remain undetected. Spatio-temporal analysis software has been previously used for multiple conditions to detect cases of disease that may have an unidentified spatial or temporal association [14–24]. One method that has gained traction as part of public health surveillance activities is the SaTScan prospective space-time permutation scan statistic [25, 26]. This analysis technique was designed to identify increases in disease across both space and time above the historic baseline to speed detection of potential clusters. While originally developed for use with syndromic data, recently it has been utilized to detect primarily community-associated clusters of Legionnaires’ disease in real time [27–29]. While promising, this method may not be ideal for detection of smaller clusters [30] or those occurring over a prolonged time [20] that may also be missed by astute clinicians or public health professionals.

We analyzed geocoded Legionnaires’ disease case data collected by the Active Bacterial Core surveillance platform, using SaTScan to simulate real-time detection of clusters across multiple states. We then compared these results to a retrospective SaTScan analysis and historic records of Legionnaires’ disease investigations to assess the strengths and limitations of the prospective space-time permutation scan statistic as an adjunct to traditional Legionnaires’ disease cluster detection methods.

## Materials and methods

Data collection and analysis activities conducted as part of the Active Bacterial Core surveillance system have been reviewed at CDC and designated as non-research.

### Active Bacterial Core surveillance (ABCs) system

Legionnaires’ disease surveillance through the ABCs platform has been previously described [31]. Briefly, laboratory-confirmed Legionnaires’ disease cases among residents of 10 surveillance areas (5 complete states and 5 defined catchments within states) representing all U.S. census regions were identified from 2011–2015. A confirmed case was defined as the isolation of *Legionella* from respiratory culture, detection of *Legionella* antigen in urine, or seroconversion (a more than fourfold rise in antibody titer between acute and convalescent sera) to *Legionella pneumophilla* serogroup 1, in addition to clinically compatible symptoms [31]. Information on patient demographics, illness symptoms, underlying conditions, disease onset date, and laboratory testing information were abstracted from medical records. Residence information was geocoded to the census tract level by participating jurisdictions and securely transmitted to CDC for analysis.

### Census data

Data from the 2010 decennial census were acquired from the U.S. Census Bureau to calculate demographic and geographic characteristics of census tracts within the ABCs Legionnaires’ disease catchment areas.

To generate the required coordinates file, TIGER/Line shape files were obtained from the U.S. Census Bureau (https://www.census.gov/geo/maps-data/data/tiger-line.html) for each of the ten ABCs states. The latitude and longitude of each census tract’s centroid were extracted. All census tracts that fell outside of the ABCs Legionnaires’ disease catchment areas were removed.

### Historical Legionnaires’ disease cluster information

Data on traditionally identified Legionnaires’ disease clusters from 2012–2015 within ABCs jurisdictions was requested from participating health departments. Clusters were defined as any grouping of cases requiring a public health investigation. Factors resulting in a full investigation likely varied by jurisdiction. Data included onset dates for the first and last confirmed case, the county where the cluster occurred, and the type of cluster (e.g., travel, healthcare, community).

### SaTScan analysis

We used SaTScan 9.4.4 in both prospective and retrospective analysis modes.

#### Prospective analysis

To simulate methods currently utilized by some health departments [20, 27], we used SAS Enterprise Guide 7.1.1 (Cary, NC) and the SaTScan prospective space-time permutation scan statistic to search for clusters. SAS EG was used to generate the SaTScan parameter files for each analysis run. Each analysis was run at the state level using a maximum temporal cluster size of 90 days (time between first and last case onset dates). This timeframe was chosen to decrease the baseline data required for analysis and to investigate methods currently in use by health departments. The time aggregation was 1 day, the minimum cluster length was 2 days, and an adjustment for day-of-the-week by space interaction was performed. We also chose to ignore clusters centered in other clusters to decrease the number of duplicate signals identified in a given timeframe. Finally, the maximum spatial cluster size was set to 6 kilometers. This was chosen based on the reported size for large community-style clusters. All other analytic options were left at their default values. Each run utilized 1 year of data. For the first run, the start and end dates were January 1, 2011, and January 1, 2012. These dates were then incremented by one day and the analysis was repeated until all 2012 data were included. The analysis was then restarted for 2013 and similarly looped until all subsequent days and years through 2015 had been included. In total, 1,460 individual analyses were performed. Case onset date was used when available; otherwise, the collection date for the confirmatory laboratory specimen was used. Signals were detected at two statistical thresholds (p<0.05 or p<0.01). These thresholds were chosen based on current SaTScan cluster detection efforts in use by New York State (unpublished) and New York City [27], respectively. Detected signals were compared to information from traditionally identified Legionnaires’ disease cluster investigations.

#### Retrospective analysis

To detect clusters retrospectively, SaTScan was run in batch mode from the command line at the state level using all 5-years of available surveillance data, 2011–2015. Other than the use of retrospective space-time analysis mode, no other settings were changed from the prospective analysis.

## Results

### ABCs Legionnaires’ disease case and outbreak surveillance data

From 2011–2015, 2527 confirmed cases of Legionnaires’ disease were identified by the ABCs network (Table 1). Census tract information was available for 2329 (92%) cases. Cases clustered geographically; 32% of census tracts contained a confirmed case.

**Table 1:** Descriptive information on confirmed LD cases by ABCs catchment area (2011–2015).

State	Statewide catchment area?	Total confirmed cases	Median cases per year (range)	Total census tracts in catchment area	Median pop per sq. mi. (range)	Median houses per sq. mi. (range)	% pop urban	Census tracts with any cases
CA	N	80	12 (11–29)	766	9190 (4232–17905)	3393 (1659–7122)	99.6%	67
CO	N	174	30 (15–54)	587	4397 (2540–6527)	1856 (986–2627)	96.3%	131
CT	Y	316	58 (57–79)	833	1961 (715–4940)	817 (266–2042)	88.0%	233
GA	N	151	30 (20–47)	699	2570 (1735–3755)	1062 (673–1671)	97.7%	131
MD	Y	714	144 (115–158)	1406	3219 (824–6493)	1277 (326–2695)	87.2%	493
MN	Y	236	51 (28–55)	1338	1463 (71–3689)	601 (32–1598)	73.3%	203
NM	Y	56	10 (9–16)	499	957 (50–3767)	410 (22–1465)	77.4%	39
NY	N	410	86 (62–104)	557	1656 (196–4530)	653 (78–2050)	74.3%	259
OR	Y	142	22 (19–49)	834	2204 (144–5000)	922 (64–2073)	81.0%	127
TN	N	248	42 (24–103)	888	1511 (421–2908)	651 (172–1316)	82.5%	109

Catchment areas varied in population density (957 persons/mi^2^ in New Mexico to 9190 persons/mi^2^ in California) and building density (410–3393 median houses/mi^2^ in the same two states). Most (87%) included counties were classified as urban according to the 2010 decennial census.

Of the 36 traditionally identified Legionnaires’ disease clusters reported by health departments (Table 2), 39% were classified as travel-associated (related to a single public accommodation), followed by community/residential (36%), and healthcare-associated (25%). Median cluster size was 2 cases (2–10). Clusters varied in length, from 2–1101 days between the first and last case onset dates.

**Table 2:** Descriptive information from traditionally identified clusters by reporting state.

State	Number of clusters	Median cases (range)	Median length (days, range)	Travel clusters	Healthcare clusters	Community/Residential
CA	1	2	3	1	0	0
CO	4	3 (2–9)	21 (2–90)	3	0	1
CT	2	7 (6–7)	33 (22–43)	0	0	2
GA	3	2 (2–10)	95 (10–180)	1	0	2
MD	11	2 (2–7)	43 (4–306)	5	1	5
MN	3	2	15 (2–24)	1	1	1
NM	0	-	-	-	-	-
NY^1^	7	2 (2–6)	83 (63–381)	0	7	0
OR	1	4	1101	1	0	0
TN^2^	4	3 (2–3)	31 (30–184)	2	0	2

^1^Data not available for entire ABCs catchment area.

^2^First and last onset date estimated.

Some outbreak investigations (19/36, 53%) had factors that made them incompatible with detection using our prospective SaTSCan analysis methodology. Most of these clusters involved travel away from the home (14/19, 74%). These could not be detected due to a lack of geocoded exposure data both from patients residing in the search area and those traveling from other jurisdictions. Other clusters in this category include those with a prolonged time between cases (5/19, 26%). These were not detectable due to the maximum temporal window chosen for this study.

### Prospective SaTScan analysis

The simulated prospective analysis identified 39 unique signals (p<0.05) (Table 3). A unique signal was defined as one that did not substantially overlap in space and time with other signals. None of the prospectively detected signals matched a traditionally identified cluster. Of the detected signals, seven met the more stringent significance threshold (p<0.01).

**Table 3:** Signals detected by prospective SaTScan analysis.

State	Signals	Median recurrence interval (range)^1^	Median cases (range)	Median Cluster radius (km,range)	# of health department clusters detected
**CA**					
p<0.05	0	-	-	-	0
p<0.01	0	-	-	-	0
**CO**					
p<0.05	3	100 (21–100)	4 (3–5)	5.76 (4.99–5.77)	0
p<0.01	0	-	-	-	0
**CT**					
p<0.05	4	40 (32–53)	3 (2–5)	4.69 (0–5.51)	0
p<0.01	0	-	-	-	0
**GA**					
p<0.05	2	53 (29–77)	3	5.56 (5.87–5.24)	0
p<0.01	0	-	-	-	0
**MD**					
p<0.05	15	81 (22–880)	4 (3–6)	4.28 (1.03–5.86)	0
p<0.01	6	145 (104–880)	5 (3–6)	4.05 (3.09–5.78)	0
**MN**					
p<0.05	4	28 (23–91)	3 (2–3)	5.13 (1.66–5.76)	0
p<0.01	0	-	-	-	0
**NM**					
p<0.05	0	-	-	-	-
p<0.01	0	-	-	-	0
**NY**					
p<0.05	4	44 (28–100)	6 (3–14)	4.86 (1.54–5.99)	0
p<0.01	0	-	-	-	0
**OR**					
p<0.05	2	101 (21–180)	3 (2–3)	4.78 (4.30–5.26)	0
p<0.01	1	180	3	4.30	0
**TN**					
p<0.05	5	37 (26–91)	3 (2–4)	3.93 (3.53–5.13)	0
p<0.01	0	-	-	-	0

^1^Reccurence interval varies inversely with the p-value and represents the number of surveillance days required to detect a similarly significant signal by chance

### Retrospective SaTScan analysis

No significant signals (p<0.05) were identified by retrospective analysis.

## Discussion

Our analysis indicates that, when standard spatial and temporal settings are utilized in a range of localities across 10 states, the SaTScan prospective space-time permutation scan statistic is not well suited for real-time detection of common types of Legionnaires’ disease clusters. This includes those associated with travel, those with a small number of cases, or those with a prolonged time between cases. Additionally, we found that signals detected by prospective SaTScan using multiple statistical thresholds (p<0.05 or p<0.01) were not identified by retrospective SaTScan or traditional methods. This discordance indicates that signals detected prospectively may not represent a true increase in cases.

During our study period, more than half of reported Legionnaires’ disease clusters health departments investigated would not have been detected using surveillance data and program settings commonly used as part of routine prospective SaTScan analyses. This was due to travel-association and a prolonged cluster length of >90 days, which are characteristics common among recognized Legionnaires’ disease clusters. Without geocoded exposure information collected from patients residing both in state and in other public health jurisdictions, space-time detection of travel clusters is unlikely. Additionally, a Legionnaires’ disease cluster detection strategy should be sensitive enough to detect as few as two cases associated with a single public accommodation (such as a hotel or healthcare facility) over a 12-month period, as that scenario should prompt a full public health investigation. These types of clusters are often associated with potable water systems, involve few cases, and may last for months or even years. Because of these factors, the addition of prospective SaTScan analysis is unlikely to improve detection of the most common types of Legionnaires’ disease clusters identified by public health authorities during our study period.

Our prospective SaTScan analysis also failed to detect traditionally identified clusters that did not involve travel or a prolonged length. This may have been due to the low number of cases associated with many of the reported clusters. This is common in Legionnaires’ disease clusters because of the low attack rate. Previous analyses have shown that SaTScan does not perform well at detecting small clusters [30], which is also the type of cluster most likely to go unrecognized by routine public health surveillance. An explosive increase in cases, such as the 2015 Bronx cluster in New York City [5], may be detected by SaTScan sooner than more traditional surveillance methods but is unlikely to go unrecognized. While timely detection of clusters is important, this benefit may decrease in areas with a lower background rate of disease, as a sharp increase in cases may be more pronounced and thus easier to detect using traditional surveillance methods.

We also found that using prospective SaTScan for cluster detection produced signals that were not found in the retrospective analysis. This strengthens our belief that these signals do not represent a true increase in disease. The ability of retrospective SaTScan analysis to detect unusual spatiotemporal associations has been shown for many other infectious diseases and non-infectious conditions such as cancer [32]. The retrospective method utilizes all available surveillance data and can identify an unusual increase in cases from any point in the study period. Conversely, prospective analysis is limited to the identification of clusters that are currently occurring. While retrospective detection of clusters is not useful for real-time surveillance purposes, neither is the detection of signals which, when analyzed in a larger historical context, are no longer statistically significant. While the reasons for the lack of concordance were not determined, we observed that no prospective SaTScan signals were identified by the retrospective method or were confirmed by traditional identification methods. Reducing the statistical threshold for the prospective analysis to p<0.01 did decrease the number of detected signals, though the lack of overlap with other detection methods remained.

The ideal scenario for SaTScan detection of Legionnaires’ disease clusters is likely a dense urban area with a high background rate of disease and a large number of aerosolizing devices such as cooling towers. Under these conditions, public health officials may struggle to determine if multiple geographically and temporally clustered cases without a known exposure source are related and thus warrant investigation. This may explain the success of SaTScan as part of routine cluster detection in locations such as New York City [33]. Other areas with a lower population density and fewer cases may rarely see the type of rapid increase in cases that SaTScan is most effective at detecting. Additionally, the lack of geocoded exposure location data from within or across jurisdictions is a significant hurdle to detection of many common cluster types. Collecting this information would be beneficial for any jurisdiction considering the adoption of geospatial cluster detection as part of routine surveillance activities.

This study had a number of limitations. First, limited descriptive information was available for clusters reported by participating states, including whether a clear exposure source was identified. Second, we only received geolocation data for patient residence. While this is a data element commonly collected by routine public health surveillance, previous studies have shown that additional location data (such as work address) can improve cluster detection [28]. Similarly, collection of possible exposure location data could aid in detection of clusters involving travel to a nearby location such as a healthcare facility. We also were unable to investigate signals produced by the prospective analysis to determine if they represent unidentified clusters. This prevents us from saying with certainty that these signals represent false positives. Finally, we did not control for possible changes in the spatial distribution of the population within each catchment area over the study period.

SaTScan software can use routinely collected surveillance data, requires minimal computer programming expertise, and is not time- or resource-intensive to operate. However, we found that the prospective space-time scan statistic utilized by SaTScan is unable to detect many Legionnaires’ disease cluster types using commonly collected geolocation data and analysis settings. Signals produced by the prospective analysis were not confirmed by other detection methods. Additional research is needed to fully understand the reasons for the lack of concordance between different SaTScan analysis methods. If this type of analysis is used for real-time cluster detection, reasonable action thresholds may help ensure that findings require a public health investigation. Spatial detection methods more tailored to the epidemiologic and environmental characteristics of Legionnaires’ disease are needed to improve cluster detection and accelerate public health action.
